# Redistribution of the astrocyte phenotypes in the medial vestibular nuclei after unilateral labyrinthectomy

**DOI:** 10.3389/fnins.2023.1146147

**Published:** 2023-06-26

**Authors:** Jie Li, Pengjun Wang, Lu-Yang Wang, Yaqin Wu, Jiping Wang, Dongzhen Yu, Zhengnong Chen, Haibo Shi, Shankai Yin

**Affiliations:** ^1^Department of Otorhinolaryngology—Head and Neck Surgery, Shanghai Sixth People’s Hospital affiliated to Shanghai Jiaotong University School of Medicine, Shanghai, China; ^2^Programs in Neurosciences & Mental Health, SickKids Research Institute and Department of Physiology, University of Toronto, Toronto, ON, Canada; ^3^Shanghai Key Laboratory of Sleep Disordered Breathing, Shanghai, China

**Keywords:** vestibular compensation, labyrinthectomy, astrocyte, single-cell RNA seq, plasticity

## Abstract

Astrocytes are highly heterogeneous and involved in different aspects of fundamental functions in the central nervous system (CNS). However, whether and how this heterogeneous population of cells reacts to the pathophysiological challenge is not well understood. To investigate the response status of astrocytes in the medial vestibular nucleus (MVN) after vestibular loss, we examined the subtypes of astrocytes in MVN using single-cell sequencing technology in a unilateral labyrinthectomy mouse model. We discovered four subtypes of astrocytes in the MVN with each displaying unique gene expression profiles. After unilateral labyrinthectomy, the proportion of the astrocytic subtypes and their transcriptional features on the ipsilateral side of the MVN differ significantly from those on the contralateral side. With new markers to detect and classify the subtypes of astrocytes in the MVN, our findings implicate potential roles of the adaptive changes of astrocyte subtypes in the early vestibular compensation following peripheral vestibular damage to reverse behavioral deficits.

## 1. Introduction

Astrocytes, one of the major glial cell populations in the central nervous system (CNS), participate in numerous functions of the CNS, including blood flow regulation, provision metabolic energy for neurons, maintaining ion balance, mediating synaptic plasticity and neurogenesis (MacVicar and Newman, 2015; Hertz and Chen, 2016; Luarte et al., 2017; Nortley and Attwell, 2017; Hussaini and Jang, 2018). In addition, astrocytes are response to many forms of brain insults and diseases, such as trauma, ischemia, epilepsy, and Alzheimer’s disease (de Lanerolle et al., 2010; Burda et al., 2016; Choudhury and Ding, 2016; Cai et al., 2017).

However, astrocytes in the CNS are highly heterogeneous. Not only the morphology and function of astrocytes differ between areas of the CNS, but also different subtypes often reside in the same brain area. Early morphological studies described two types of central astrocytes: fibrous astrocytes in white matter and protoplasmic astrocytes in gray matter (Andriezen, 1893). Fibrous astrocytes extend long, thin, and less branched processes, while protoplasmic astrocytes have short, thick and fine-branched processes. A number of other astrocytes with distinct morphological forms such as Bergmann glia specific in cerebellum and Müller glia of the retina have also been reported (Grosche et al., 2002; Bringmann et al., 2006). Furthermore, gene and protein expression differs between astrocytic subtypes. For example, glutamate transporter EAAT2 is expressed at a higher level in the cerebral cortex, hippocampus and striatum, while EAAT1 is preferentially expressed in the cerebellum (Lehre et al., 1995). Synaptogenic modulator *Sparc* is enriched in the hypothalamus/thalamus but expressed at a low level in the cortex/hippocampus (Morel et al., 2017). These studies indicate that regional molecular astrocyte diversity is associated with the functional features of astrocytes. Previous studies have considered a number of genes as astrocyte-specific markers including *Aldh1l1, Aqp4, Gjb1, Gja1* and *Gli1*(Pfrieger and Slezak, 2012). Despite these optional molecular markers, there are currently no canonical molecular markers that can fully define the subtype of astrocytes because none of these molecules is expressed by all types of astrocytes, and some of these markers (*Aldh1l1, Gli1*) are also identified in cells with stem cell properties at embryonic and adult stage. Nevertheless, the glial fibrillary acidic protein (GFAP), an intermediate filament protein expressed in astrocytes and radial glia, is ubiquitously considered as a classic marker despite not all astrocytes express detectable level of GFAP (Walz and Lang, 1998; Walz, 2000) and often used to distinguish astrocytes from other cells of the central nervous system (Sofroniew and Vinters, 2010). Moreover, GFAP expression is usually upregulated in reactive astrocytes which are activated under injury or disease conditions (Sofroniew, 2009; Pekny et al., 2019).

Heterogeneity of astrocytes in the CNS may allow their diverse responses to different pathological processes by alterations in the gene expression profile and functions, facilitating adaptation to the local tissue environment of the CNS. For example, in the animal model of Alzheimer’s disease, the patterns of GFAP expression varies among brain areas and the different stage of the disease, indicating the concomitant astrocytic atrophy and astrogliosis during the neurodegenerative process (Olabarria et al., 2010). Decreased AQP4 protein levels of astrocytes was detected by immunohistochemical method in the hippocampus early in the epilepsy model (Lee et al., 2012). Although there is circumstantial evidence showing a proliferation of astrocytes with high level of GFAP expression in the medial vestibular nucleus (MVN) of cat after unilateral vestibular neurectomy (Dutheil et al., 2013), whether and how different astrocytes in the MVN are engaged remain unknow. In the present study, we discovered 4 subtypes of astrocytes in the MVN and described their response to vestibular loss at single-cell resolution.

## 2. Materials and methods

### 2.1. Unilateral labyrinthectomy

C57BL/6J mice of P20 were anesthetized with pentobarbital sodium (40 mg/kg). A post-auricular incision was made in the right ear to expose the tympanic bulla. The malleus and incus were removed under a microscope. The stapedial artery was exposed and coagulated. Then the oval window was open and expand. The utricular maculae, saccular maculae and semicircular canal ampulla were destroyed and vaporized with electrotome. The cavity was rinsed with 100% ethanol and filled with gelfoam and the skin incision was sutured. The animals were sacrificed 1 day after unilateral labyrinthectomy (UL) for the next experiment. In the control group, only the tympanic bulla was opened, but UL was not performed.

### 2.2. Immunofluorescence

Mice were anesthetized with sodium pentobarbital, and transcardially perfused with 20 mL of 4% paraformaldehyde. The brain was removed and immersed in the same fixative at 4°C for 12 h. The tissue was embedded in paraffin and cut with a microtome (CV 5030; Leica, Germany) into 4um sections. The sections were incubated in 10% bovine serum in PBS containing 0.1% Triton X-100 for 30 min. Anti-mouse GFAP primary antibody (1:500; Abcam) was added to the slices, incubating at 4°C overnight. After washing, the sections were added with a rabbit secondary antibody (1:1000; Abcam) conjugated to AlexaFluor 488 and kept in the dark at room temperature for 2 h. DAPI was added to stain the cell nuclei. Micrographs were taken with a fluorescence microscope.

### 2.3. Quantification and statistical analysis

Pictures per section were taken with a 20× objective, and data were collected from 3 animals of each group. The integrated fluorescence intensity of GFAP on each slice was measured by Image J software (1.8.0) and was averaged by dividing the area of MVN (Orr et al., 2020). The statistics were performed using one-way ANOVA with Tukey HSD *post hoc* test. *p*-values <0.05 were considered statistically significant.

### 2.4. Single-cell RNA sequencing and data analysis

After anesthetization, brains of mice were removed from the skull bone. Brain slices of 500 μm containing MVN tissue were made with a vibration microtome. MVN tissues were dissected under a microscope and dissociated into single-cell suspension according to 10X genomics chromium sample preparation protocol. Single-cells with barcoded beads were captured into nanoliter-sized droplets using a microfluidic device. Then cDNA library was constructed and sequenced on Illumina HiSeqX10. Cell clustering analysis was performed using the R package Seurat (2.3.4) and visualized by 3D and 2D t-stochastic neighbor embedding (t-SNE) (Zeisel et al., 2015). The R package Monocle (2.12.0) was applied for pseudotime analysis (Trapnell et al., 2014).

## 3. Results

**Figure 1:**
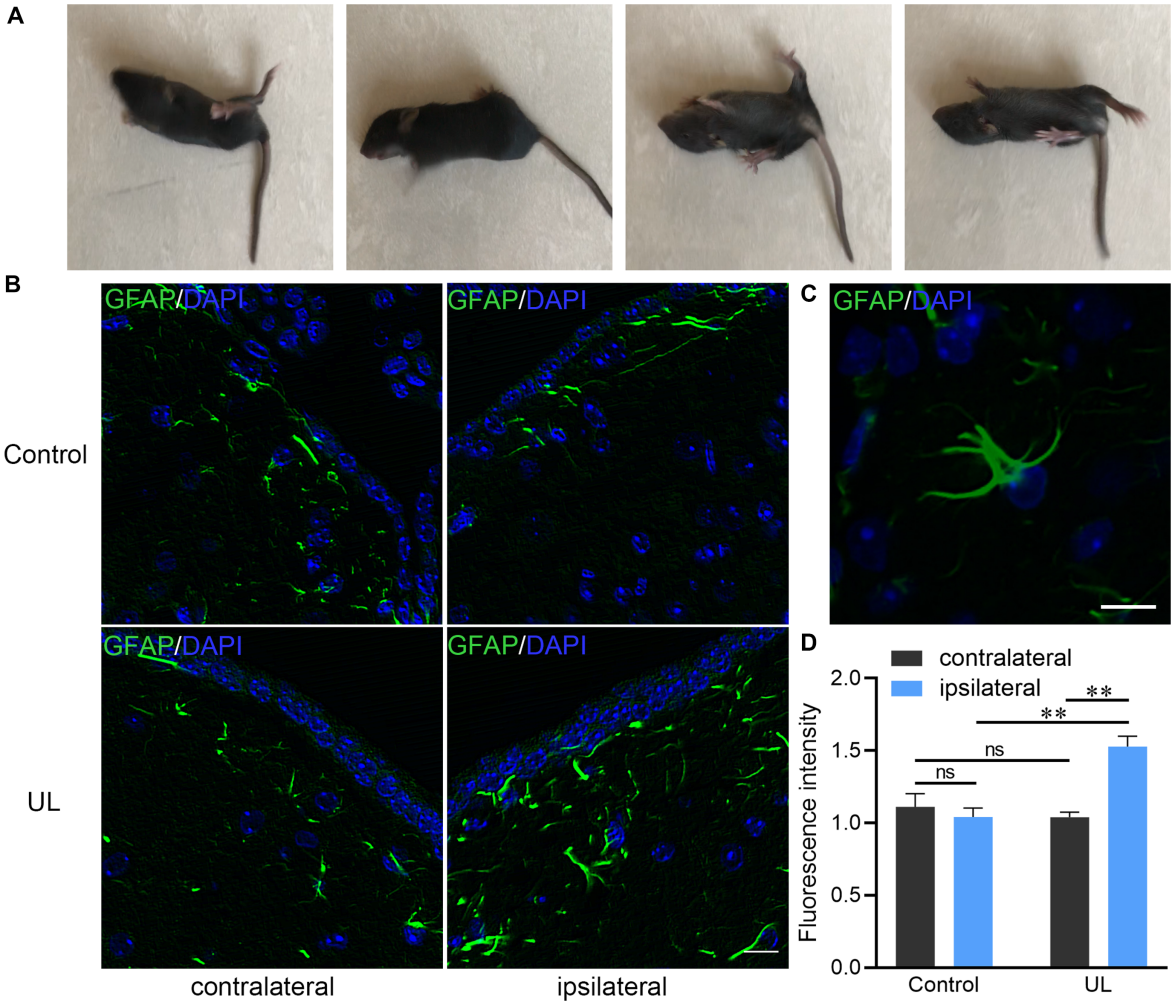
Changes of behavior and GFAP immunoreactive astrocytes after unilateral labyrinthectomy (UL). **(A)** The mouse could not maintain balance on the smooth ground and rolled towards the affected side 1 day after UL. **(B)** Photomicrographs double-labeled for GFAP (green) and DAPI (blue) in the MVN after sham operations and after UL. Scale bar, 10um. **(C)** A representative astrocyte in the MVN with several GFAP (+) processes. Scale bar, 10um. **(D)** The ipsilateral MVN in the UL group showing much higher levels of GFAP immunoreactive fluorescence intensity than the contralateral MVN as well as the ipsilateral MVN in the control group. One-way ANOVA with the Tukey HSD *post hoc* test. ^**^Indicates *p* < 0.01.

The manifestation caused by UL such as head tilting toward the ipsilateral side and rotation of the body were observed in all mice underwent UL (Figure 1A). The bilateral MVN in the sham operation group showed the same pattern of GFAP expression, low level of GFAP immunostaining of astrocytic processes. In contrast, in the UL group, the ipsilateral MVN presented much higher levels of GFAP immunoactivity than the contralateral MVN after UL (Figure 1B). GFAP immunopositive processes were observed in a representative astrocyte in the MVN (Figure 1C). The average fluorescent intensity of GFAP expression level is higher in the ipsilateral MVN than that in the contralateral MVN and the control group (Figure 1D).

**Figure 2:**
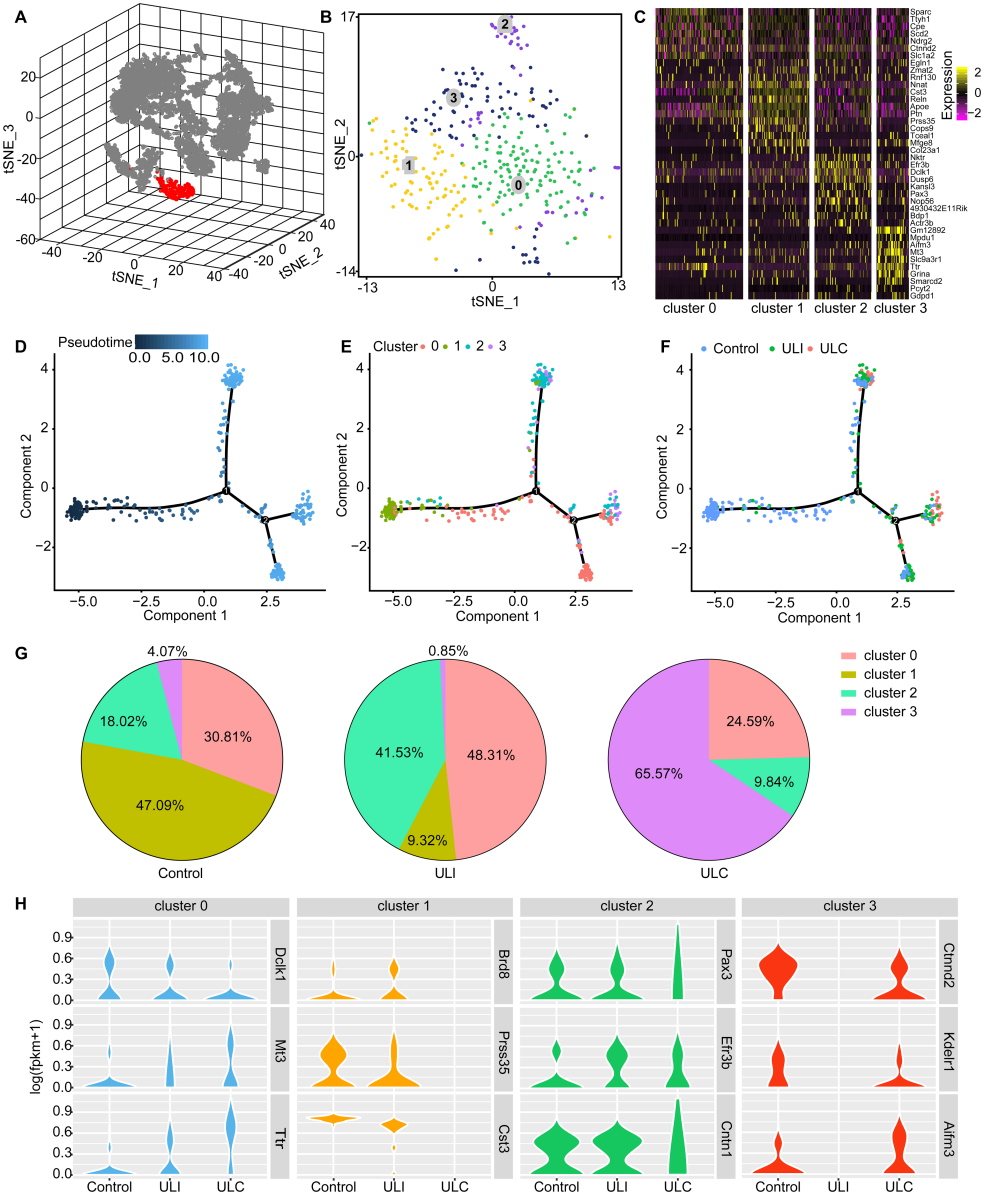
Identification of 4 subtypes of astrocytes in the mouse MVN and UL-induced subtype redistribution. **(A)** 3D t-SNE diagram shows astrocytes and other cell populations in the MVN. Astrocytes were identified based on the expression of the marker genes *Agt, Slc6a11, Gja1, Gjb6, Fgfr3*, and *Aqp4*. Astrocytes (red), non-astrocytes (gray). **(B)** 2D t-SNE diagram visualizes the 4 subtypes of astrocytes in graph **(A)** with different cell subtypes being color-coded. **(C)** Heatmap showing the subtype-specific expression of marker genes across the 4 subtypes of astrocytes in the MVN. Rows indicate individual genes and columns represent individual cells. **(D–F)** Pseudotime analysis reveals the distribution of the 4 astrocytic subtypes along the pseudotime trajectory in the control and UL group. **(D)** The time sequence of the pseudotime trajectory was color-coded. **(E)** The distribution of 4 subtypes of astrocytes along the pseudotime trajectory. Each astrocytic subtype is color-coded. **(F)** Astrocytes from the control group and the bilateral MVN from the UL group were showed in the pseudotime trajectory. These 3 groups of cells are color-coded. ULI (ipsilateral MVN of UL group), ULC (contralateral MVN of UL group). **(G)** The pie charts show the proportion of each subtype of astrocytes in the control MVN (left), the ipsilateral MVN (middle), and the contralateral MVN (right). **(H)** UL-induced transcriptional alterations in different astrocyte subclusters. Violin plots showing representative differentially expressed genes in different astrocytic subtypes caused by UL. Gene expression level is illustrated on a log scale.

To address the heterogeneity of astrocytes in the MVN and their adaptive changes, we performed single-cell RNA sequencing to characterize the transcriptional features of the astrocytes responding to UL. For clustering analysis, we combined the cells from 10 control and 11 UL mice to increase cell number. Based on the expression of the enriched astrocyte makers *Agt*, *Slc6a11*, *Gja1*, *Gjb6*, *Fgfr3*, and *Aqp4*, which code the proteins angiotensinogen, GAT-3, connexin 43, connexin 30 and aquaporin-4, respectively (Pringle et al., 2003; Brenner, 2014; Almad et al., 2016; Boddum et al., 2016; Ikeshima-Kataoka, 2016; De Bock et al., 2017; Pannasch et al., 2019), we isolated 351 astrocytes from all the captured cells in the MVN (Figure 2A). Our clustering analysis further subclustered the astrocytes to 4 major subtypes based on different features of their transcriptional profiles (Supplementary Table 1) and visualized their distribution through 2D t-SNE plot (Figure 2B). We generated heatmap of gene expression and identified marker genes for each of the astrocytic subclusters (Figure 2C). The four astrocytic subtypes were distinguished from each other by the expression of differential gene markers. For example, *Sparc* (osteonectin) represents astrocytes of cluster 0, while *Nnat* (neuronatin) indicates astrocytes of cluster 1. *Nktr* (natural killer tumor recognition protein) and *Slc9a3r1* (Na(+)/H(+) exchange regulatory cofactor) serve as markers of cluster 2 and cluster 3, respectively. To investigate the astrocytic alterations induced by UL, we next compared the components of astrocytic subclusters in the control MVN and bilateral MVN in the UL group using pseudotime analysis (Figures 2D–F). The direction of pseudotime trajectory was illustrated with color-coding (Figure 2D). The unequal distribution of 4 astrocytic subtypes was illustrated along the pseudotime line. The majority of subcluster 1 astrocytes located in the start on the line while other cell subtypes were mainly on the ends of each branch (Figure 2E). Likewise, astrocytes from the MVN of control and UL group were asymmetrically distributed along the pseudotime line. Evidently, we found that the major astrocytes of the control group located in the start of the line, consistent with the distribution of cells of cluster 1, while other astrocytic subtypes spread on different ends of the branches (Figure 2F).

To further characterize the alterations of the astrocytic subtypes, we calculated the components of subclusters in the MVN of control group and the bilateral MVN of the UL group. Pie plots showed the distribution of astrocytic subtypes in the control MVN, the ipsilateral MVN and the contralateral MVN of the UL group. These plots unveil a robust redistribution of astrocyte phenotypes post-UL in both ipsilateral and contralateral sides with cluster 0 and 2 being increased on the ipsilateral side but decreased on the contralateral side, whereas cluster 3 showed opposite change and cluster 1 decreased bilaterally (Figure 2G), implicating bilateral adaptations of astrocytes in MVN after UL. Further, the transcriptional response to UL across the 4 astrocytic subtypes of MVN by comparing gene expression profiles was examined between the control group and the UL group. The results uncovered that acute vestibular loss has different impacts on gene expression of each astrocyte subtype, such as *Dclk1*, *Mt3*, *Ttr* in cluster 0, *Brd8*, *Prss35*, *Cst3* in cluster 1, *Pax3*, *Efr3b*, *Cntn1* in cluster 2, and *Ctnnd2*, *Kdelr1*, *Aifm3* in cluster3. These differentially expressed genes contribute to transcription (*Brd8, Pax3*), neurogenesis and synaptogenesis (*Dclk1, Ctnnd2*), neuroprotective response (*Mt3, Ttr*), cell death (*Cst3, Aifm3*) and cellular localization (*Efr3b, Kdelr1*). Aside from a redistribution of subtypes that have opposite trajectories in proportions between ipsilateral and contralateral sides, the gene expression was drastically altered mainly in astrocytes of cluster 1 and cluster 3, suggesting that these two astrocytic subtypes are the main responsive cell types involved in regulating the early vestibular compensation (Figure 2H).

## 4. Discussion

In the current study, we discovered 4 major phenotypic astrocytes in MVN and demonstrated their differential responses to the acute vestibular loss in the mice MVN by analyzing transcriptional markers enriched in astrocytes of different cellular populations. The maturation perturbation could be excluded to a large extent by comparing the UL group with the control group. These gene markers will provide valid molecular signatures to classify different astrocytes and facilitate future vestibular research *via* genetic labeling and specific manipulations based on astrocytic subtypes. More importantly, such a classification provides cellular substrates to investigate how different subtypes trigger or regulate the vestibular compensation (VC) which reflects an innate recovery of vestibular functions essential for postural, locomotor, and oculomotor functions after peripheral vestibular damage. VC depends mainly on the plasticity in the CNS because of the limited regenerative capacity in the peripheral vestibular epithelium (Kawamoto et al., 2009; Lacour et al., 2016). However, about 20%–30% patients with unilateral vestibular loss are poorly compensated, which indicates the recovery of vestibular function does not happen or is defective (Macdougall and Curthoys, 2012). Therefore, facilitation of VC is considered as an effective strategy to ameliorate vestibular disorders. Although the mechanisms underlying VC still remains elusive, emerging evidence implicates the changes of neuronal intrinsic properties, regulation of neurotransmitter receptors, neurogenesis and glial proliferation (Straka et al., 2005; Tighilet et al., 2007; Zhou et al., 2016; Chen et al., 2019). Previous studies that focused mainly on the alteration of GFAP immunoreactive astrocytes in the MVN after vestibular loss indicated that astrocytes contributed to the process of VC (Campos-Torres et al., 2005; Tighilet and Chabbert, 2019). However, the challenge is that in the CNS there is a large population of astrocytes with diverse function which are not always GFAP immunoreactive (Kimelberg, 2004). Although an increase of GFAP immunostaining was confirmed in the ipsilateral MVN of mice in our experiments, we suggest other gene markers including *Agt*, *Slc6a11*, *Gja1*, *Gjb6*, *Fgfr3*, and *Aqp4* to complement the identification classification of the astrocytes. These markers well illustrate the same astrocytic cell populations (Figure 2A).

In this study, we first demonstrated that the heterogeneity of transcriptomic features exists among the diverse astrocytes in the MVN. Previous studies by detecting the astroglia markers hGFAP-GFP expression, and GFAP and S100β immunostaining reported that there are 9 subtypes of astrocytes resident in different area of mammalian CNS and only the velate astrocytic subtype locates in the brainstem (Emsley and Macklis, 2006). However, these limited markers are not adequate to distinguish the subtypes of astrocyte in the brainstem. By high-throughput single-cell RNA sequencing method, we further classified the astrocytes in the MVN and comprehensively studied the transcriptomic features within each of the astrocytic subclusters. Previous studies on single-cell transcriptomics observed molecularly distinct and region-specific astrocyte types in the mouse central nervous system under normal conditions (Zeisel et al., 2018; Batiuk et al., 2020; Bayraktar et al., 2020). These subtypes could be correlated with neurotransmission and synaptogenesis (Zeisel et al., 2018; Miller et al., 2019). Batiuk et al. (2020) identified five astrocyte subtypes in the mouse cortex and hippocampus, while in our study, we classified the astrocytes in MVN into four subtypes. Consistent with the previous research, all subtypes expressed *Agt, Apq4 and Sox9*. A recent study discovered that astrocytes display diverse reactive phenotypes according to their region-specific homeostatic identities in response to different brain insults and a common gene set (*Gfap, Vim, Serpina3n*) was involved (Makarava et al., 2023). Different from these insult models, labyrinthectomy does not cause direct damage to brain tissue. In the functional deafferentation animals, we observed an increased GAFP immunoreactivity in the ipsilateral MVN and a higher proportion of cluster 0 associated with synaptogenesis.

Investigation of the altered composition of astrocytic subtypes and their differential expressed genes induced by UL in the MVN is important for understanding the function of this important brain region in the process of VC. In addition to the difference astrocytic subclusters between the control and UL group, the different gene expression patterns of astroglia between the bilateral MVN in the UL group was clearly observed in our study, indicating the balance of activity in the bilateral MVN was disturbed by UL. Previous studies have demonstrated that inhibitory neurons in the ipsilateral developed a sustained hyperactivity immediately after UL, and resulted in inhibitory overtone onto contralateral neurons *via* commissural projection at early stage of VC (Guilding and Dutia, 2005; Bergquist et al., 2008; Chen et al., 2019). Our finding that opposite changes in the proportion of Cluster 0 and 2 astrocytes between ipsilateral and contralateral sides parallels the imbalance of neural activity, suggesting astrocytes may be engaged in fostering adaptative changes of neural circuits to achieve VC.

In conclusion, our study elucidates the classification of astrocytes in the mouse MVN and delineates the detailed changes of astrocytic subtypes following UL by analyzing the transcriptome landscape of astroglia at single-cell resolution. Furthermore, by comparing different expressed genes, we identified several candidate molecular markers of astrocytes involved in the early process of VC. Asymmetric changes in the proportion of different astrocyte subtypes and their gene expression between ipsilateral and contralateral sides are likely crucial for remodeling the neural circuit for vestibular compensation following peripheral vestibular damage. Our findings pave the way for future mechanistic and translational studies on the functions of different astrocyte subtypes for rectifying behavioral deficits associated the vestibular disorders.

## Data availability statement

The datasets presented in this study can be found in online repositories. The names of the repository/repositories and accession number(s) can be found at: https://www.ncbi.nlm.nih.gov/geo/query/acc.cgi?acc=GSE143701.

## Ethics statement

The animal study was reviewed and approved by the Sixth People’s Hospital Affiliated to Shanghai Jiao Tong University (Approval no: 2018-KY-003). Written informed consent was obtained from the owners for the participation of their animals in this study.

## Author contributions

PW conceived and designed the study and performed experiments. JL analyzed the data and drafted the manuscript. HS and ZC helped to design the study and corrected the manuscript. L-YW and SY critically revised the manuscript for important content. L-YW helped to interpret results. JW, YW, and DY helped to draft the manuscript and collected the data. All authors contributed to the article and approved the submitted version.

## Funding

This study was funded by National Natural Science Foundation of China (nos. 82171140 and 82020108008), and the Project of Shanghai Jiao Tong University Medicine Science and Engineering Interdisciplinary Foundation (no. YG2021QN106).

## Conflict of interest

The authors declare that the research was conducted in the absence of any commercial or financial relationships that could be construed as a potential conflict of interest.

## Publisher’s note

All claims expressed in this article are solely those of the authors and do not necessarily represent those of their affiliated organizations, or those of the publisher, the editors and the reviewers. Any product that may be evaluated in this article, or claim that may be made by its manufacturer, is not guaranteed or endorsed by the publisher.

## Supplementary material

The Supplementary material for this article can be found online at: https://www.frontiersin.org/articles/10.3389/fnins.2023.1146147/full#supplementary-material

## References

[ref1] Almad A. A. Doreswamy A. Gross S. K. Richard J. P. Huo Y. Haughey N.. (2016). Connexin 43 in astrocytes contributes to motor neuron toxicity in amyotrophic lateral sclerosis. Glia 64, 1154–1169. doi: 10.1002/glia.22989, PMID: 27083773 PMC5635605

[ref2] Andriezen W. L. (1893). The neuroglia elements in the human brain. Br. Med. J. 2, 227–230. doi: 10.1136/bmj.2.1700.227, PMID: 20754383 PMC2422013

[ref3] Batiuk M. Y. Martirosyan A. Wahis J. de Vin F. Marneffe C. Kusserow C.. (2020). Identification of region-specific astrocyte subtypes at single cell resolution. Nat. Commun. 11, 1220–1234. doi: 10.1038/s41467-019-14198-8, PMID: 32139688 PMC7058027

[ref4] Bayraktar O. A. Bartels T. Holmqvist S. Kleshchevnikov V. Martirosyan A. Polioudakis D.. (2020). Astrocyte layers in the mammalian cerebral cortex revealed by a single-cell in situ transcriptomic map. Nat. Neurosci. 23, 500–509. doi: 10.1038/s41593-020-0602-1, PMID: 32203496 PMC7116562

[ref5] Bergquist F. Ludwig M. Dutia M. B. (2008). Role of the commissural inhibitory system in vestibular compensation in the rat. J. Physiol. 586, 4441–4452. doi: 10.1113/jphysiol.2008.155291, PMID: 18635647 PMC2614028

[ref6] Boddum K. Jensen T. P. Magloire V. Kristiansen U. Rusakov D. A. Pavlov I.. (2016). Astrocytic GABA transporter activity modulates excitatory neurotransmission. Nat. Commun. 7:13572. doi: 10.1038/ncomms13572, PMID: 27886179 PMC5133667

[ref7] Brenner M. (2014). Role of GFAP in CNS injuries. Neurosci. Lett. 565, 7–13. doi: 10.1016/j.neulet.2014.01.055, PMID: 24508671 PMC4049287

[ref8] Bringmann A. Pannicke T. Grosche J. Francke M. Wiedemann P. Skatchkov S. N.. (2006). Muller cells in the healthy and diseased retina. Prog. Retin. Eye Res. 25, 397–424. doi: 10.1016/j.preteyeres.2006.05.003 16839797

[ref9] Burda J. E. Bernstein A. M. Sofroniew M. V. (2016). Astrocyte roles in traumatic brain injury. Exp. Neurol. 275, 305–315. doi: 10.1016/j.expneurol.2015.03.020, PMID: 25828533 PMC4586307

[ref10] Cai Z. Wan C. Q. Liu Z. (2017). Astrocyte and Alzheimer’s disease. J. Neurol. 264, 2068–2074. doi: 10.1007/s00415-017-8593-x 28821953

[ref11] Campos-Torres A. Touret M. Vidal P. P. Barnum S. de Waele C. (2005). The differential response of astrocytes within the vestibular and cochlear nuclei following unilateral labyrinthectomy or vestibular afferent activity blockade by transtympanic tetrodotoxin injection in the rat. Neuroscience 130, 853–865. doi: 10.1016/j.neuroscience.2004.08.052, PMID: 15652984

[ref12] Chen Z. P. Zhang X. Y. Peng S. Y. Yang Z. Q. Wang Y. B. Zhang Y. X.. (2019). Histamine H1 receptor contributes to vestibular compensation. J. Neurosci. 39, 420–433. doi: 10.1523/JNEUROSCI.1350-18.2018, PMID: 30413645 PMC6335742

[ref13] Choudhury G. R. Ding S. (2016). Reactive astrocytes and therapeutic potential in focal ischemic stroke. Neurobiol. Dis. 85, 234–244. doi: 10.1016/j.nbd.2015.05.003, PMID: 25982835 PMC4644522

[ref14] De Bock M. Leybaert L. Giaume C. (2017). Connexin channels at the Glio-vascular Interface: gatekeepers of the brain. Neurochem. Res. 42, 2519–2536. doi: 10.1007/s11064-017-2313-x, PMID: 28634726

[ref15] de Lanerolle N. C. Lee T. S. Spencer D. D. (2010). Astrocytes and epilepsy. Neurotherapeutics 7, 424–438. doi: 10.1016/j.nurt.2010.08.002, PMID: 20880506 PMC5084304

[ref16] Dutheil S. Escoffier G. Gharbi A. Watabe I. Tighilet B. (2013). GABA(a) receptor agonist and antagonist alter vestibular compensation and different steps of reactive neurogenesis in deafferented vestibular nuclei of adult cats. J. Neurosci. 33, 15555–15566. doi: 10.1523/JNEUROSCI.5691-12.2013, PMID: 24068822 PMC6618455

[ref17] Emsley J. G. Macklis J. D. (2006). Astroglial heterogeneity closely reflects the neuronal-defined anatomy of the adult murine CNS. Neuron Glia Biol. 2, 175–186. doi: 10.1017/S1740925X06000202, PMID: 17356684 PMC1820889

[ref18] Grosche J. Kettenmann H. Reichenbach A. (2002). Bergmann glial cells form distinct morphological structures to interact with cerebellar neurons. J. Neurosci. Res. 68, 138–149. doi: 10.1002/jnr.10197, PMID: 11948659

[ref19] Guilding C. Dutia M. B. (2005). Early and late changes in vestibular neuronal excitability after deafferentation. Neuroreport 16, 1415–1418. doi: 10.1097/01.wnr.0000176519.42218.a6, PMID: 16110261

[ref20] Hertz L. Chen Y. (2016). Importance of astrocytes for potassium ion (K^+^) homeostasis in brain and glial effects of K^+^ and its transporters on learning. Neurosci. Biobehav. Rev. 71, 484–505. doi: 10.1016/j.neubiorev.2016.09.018, PMID: 27693230

[ref21] Hussaini S. M. Q. Jang M. H. (2018). New roles for old glue: astrocyte function in synaptic plasticity and neurological disorders. Int. Neurourol. J. 22, S106–S114. doi: 10.5213/inj.1836214.107, PMID: 30396259 PMC6234728

[ref22] Ikeshima-Kataoka H. (2016). Neuroimmunological implications of AQP4 in astrocytes. Int. J. Mol. Sci. 17:1306. doi: 10.3390/ijms17081306, PMID: 27517922 PMC5000703

[ref23] Kawamoto K. Izumikawa M. Beyer L. A. Atkin G. M. Raphael Y. (2009). Spontaneous hair cell regeneration in the mouse utricle following gentamicin ototoxicity. Hear. Res. 247, 17–26. doi: 10.1016/j.heares.2008.08.010, PMID: 18809482 PMC2905733

[ref24] Kimelberg H. K. (2004). The problem of astrocyte identity. Neurochem. Int. 45, 191–202. doi: 10.1016/j.neuint.2003.08.015, PMID: 15145537

[ref25] Lacour M. Helmchen C. Vidal P. P. (2016). Vestibular compensation: the neuro-otologist's best friend. J. Neurol. 263, S54–S64. doi: 10.1007/s00415-015-7903-4. 27083885 PMC4833803

[ref26] Lee D. J. Hsu M. S. Seldin M. M. Arellano J. L. Binder D. K. (2012). Decreased expression of the glial water channel aquaporin-4 in the intrahippocampal kainic acid model of epileptogenesis. Exp. Neurol. 235, 246–255. doi: 10.1016/j.expneurol.2012.02.002, PMID: 22361023 PMC3334411

[ref27] Lehre K. P. Levy L. M. Ottersen O. P. Storm-Mathisen J. Danbolt N. C. (1995). Differential expression of two glial glutamate transporters in the rat brain: quantitative and immunocytochemical observations. J. Neurosci. 15, 1835–1853. doi: 10.1523/JNEUROSCI.15-03-01835, PMID: 7891138 PMC6578153

[ref28] Luarte A. Cisternas P. Caviedes A. Batiz L. F. Lafourcade C. Wyneken U.. (2017). Astrocytes at the hub of the stress response: potential modulation of neurogenesis by miRNAs in astrocyte-derived exosomes. Stem Cells Int. 2017:1719050. doi: 10.1155/2017/1719050 29081809 PMC5610870

[ref29] Macdougall H. G. Curthoys I. S. (2012). Plasticity during vestibular compensation: the role of saccades. Front. Neurol. 3:21. doi: 10.3389/fneur.2012.00021 22403569 PMC3289127

[ref30] MacVicar B. A. Newman E. A. (2015). Astrocyte regulation of blood flow in the brain. Cold Spring Harb. Perspect. Biol. 7:a020388. doi: 10.1101/cshperspect.a020388, PMID: 25818565 PMC4448617

[ref31] Makarava N. Mychko O. Molesworth K. Chang C. Y. J. Henry R. J. Tsymbalyuk N.. (2023) Region-specific homeostatic identity of astrocytes is essential for defining their reactive phenotypes following pathological insults. *biorxiv* Available at: 10.1101/2023.02.01.526708. PMC10486627 37681904

[ref32] Miller S. J. Philips T. Kim N. Dastgheyb R. Chen Z. Hsieh Y. C.. (2019). Molecularly defined cortical astroglia subpopulation modulates neurons via secretion of Norrin. Nat. Neurosci. 22, 741–752. doi: 10.1038/s41593-019-0366-7, PMID: 30936556 PMC6551209

[ref33] Morel L. Chiang M. S. R. Higashimori H. Shoneye T. Iyer L. K. Yelick J.. (2017). Molecular and functional properties of regional astrocytes in the adult brain. J. Neurosci. 37, 8706–8717. doi: 10.1523/JNEUROSCI.3956-16.2017, PMID: 28821665 PMC5588463

[ref34] Nortley R. Attwell D. (2017). Control of brain energy supply by astrocytes. Curr. Opin. Neurobiol. 47, 80–85. doi: 10.1016/j.conb.2017.09.012 29054039

[ref35] Olabarria M. Noristani H. N. Verkhratsky A. Rodriguez J. J. (2010). Concomitant astroglial atrophy and astrogliosis in a triple transgenic animal model of Alzheimer's disease. Glia 58, NA–838. doi: 10.1002/glia.20967 20140958

[ref36] Orr B. O. Hauswirth A. G. Celona B. Fetter R. D. Zunino G. Kvon E. Z.. (2020). Presynaptic homeostasis opposes disease progression in mouse models of ALS-like degeneration: evidence for homeostatic neuroprotection. Neuron 107, 95–111.e6. doi: 10.1016/j.neuron.2020.04.009, PMID: 32380032 PMC7529479

[ref37] Pannasch U. Dossi E. Ezan P. Rouach N. (2019). Astroglial Cx30 sustains neuronal population bursts independently of gap-junction mediated biochemical coupling. Glia 67, 1104–1112. doi: 10.1002/glia.23591 30794327 PMC6519011

[ref38] Pekny M. Wilhelmsson U. Tatlisumak T. Pekna M. (2019). Astrocyte activation and reactive gliosis-a new target in stroke? Neurosci. Lett. 689, 45–55. doi: 10.1016/j.neulet.2018.07.021, PMID: 30025833

[ref39] Pfrieger F. W. Slezak M. (2012). Genetic approaches to study glial cells in the rodent brain. Glia 60, 681–701. doi: 10.1002/glia.22283 22162024

[ref40] Pringle N. P. Yu W. P. Howell M. Colvin J. S. Ornitz D. M. Richardson W. D. (2003). Fgfr3 expression by astrocytes and their precursors: evidence that astrocytes and oligodendrocytes originate in distinct neuroepithelial domains. Development 130, 93–102. doi: 10.1242/dev.00184 12441294

[ref41] Sofroniew M. V. (2009). Molecular dissection of reactive astrogliosis and glial scar formation. Trends Neurosci. 32, 638–647. doi: 10.1016/j.tins.2009.08.002, PMID: 19782411 PMC2787735

[ref42] Sofroniew M. V. Vinters H. V. (2010). Astrocytes: biology and pathology. Acta Neuropathol. 119, 7–35. doi: 10.1007/s00401-009-0619-8, PMID: 20012068 PMC2799634

[ref43] Straka H. Vibert N. Vidal P. P. Moore L. E. Dutia M. B. (2005). Intrinsic membrane properties of vertebrate vestibular neurons: function, development and plasticity. Prog. Neurobiol. 76, 349–392. doi: 10.1016/j.pneurobio.2005.10.002, PMID: 16263204

[ref44] Tighilet B. Brezun J. M. Sylvie G. D. Gaubert C. Lacour M. (2007). New neurons in the vestibular nuclei complex after unilateral vestibular neurectomy in the adult cat. Eur. J. Neurosci. 25, 47–58. doi: 10.1111/j.1460-9568.2006.05267.x, PMID: 17241266

[ref45] Tighilet B. Chabbert C. (2019). Adult neurogenesis promotes balance recovery after vestibular loss. Prog. Neurobiol. 174, 28–35. doi: 10.1016/j.pneurobio.2019.01.001, PMID: 30658127

[ref46] Trapnell C. Cacchiarelli D. Grimsby J. Pokharel P. Li S. Morse M.. (2014). The dynamics and regulators of cell fate decisions are revealed by pseudotemporal ordering of single cells. Nat. Biotechnol. 32, 381–386. doi: 10.1038/nbt.2859, PMID: 24658644 PMC4122333

[ref47] Walz W. (2000). Controversy surrounding the existence of discrete functional classes of astrocytes in adult gray matter. Glia 31, 95–103. doi: 10.1002/1098-1136(200008)31:2<95::aid-glia10>3.0.co;2-6, PMID: 10878596

[ref48] Walz W. Lang M. K. (1998). Immunocytochemical evidence for a distinct GFAP-negative subpopulation of astrocytes in the adult rat hippocampus. Neurosci. Lett. 257, 127–130. doi: 10.1016/s0304-3940(98)00813-1, PMID: 9870336

[ref49] Zeisel A. Hochgerner H. Lönnerberg P. Johnsson A. Memic F. van der Zwan J.. (2018). Molecular architecture of the mouse nervous system. Cells 174, 999–1014.e22. doi: 10.1016/j.cell.2018.06.021, PMID: 30096314 PMC6086934

[ref50] Zeisel A. Munoz-Manchado A. B. Codeluppi S. Lonnerberg P. La Manno G. Jureus A.. (2015). Brain structure. Cell types in the mouse cortex and hippocampus revealed by single-cell RNA-seq. Science 347, 1138–1142. doi: 10.1126/science.aaa1934, PMID: 25700174

[ref51] Zhou W. Zhou L. Q. Shi H. Leng Y. M. Liu B. Zhang S. L.. (2016). Expression of glycine receptors and gephyrin in rat medial vestibular nuclei and flocculi following unilateral labyrinthectomy. Int. J. Mol. Med. 38, 1481–1489. doi: 10.3892/ijmm.2016.2753, PMID: 28026001 PMC5065303

